# Supplementary material for: Geographic-style maps with a local novelty distance help navigate in the materials space
Source: Sci Rep. 2025 Jul 29;15:27588. doi: 10.1038/s41598-025-10672-0 (PMC12307791; doi:10.1038/s41598-025-10672-0)

Appendix A Extra examples and navigation maps

This appendix includes extra examples of invariant computations for 5 perovskites in addition to 5 simple crystals in Fig. 3, see corresponding entries from the MP in Table A1, instructions for running the Python code for all invariants, and high-resolution navigation maps. The zip folder with supplementary information includes the Python code and tables with PDD and PDA matrices for the 5+5 example crystals. Fig. A1 and Table A2 compare unweighted (based on atomic centers) and weighted versions (including atomic masses) of invariants. The atomic masses generally increase EMD distances but the geometry of atomic centers already captures chemistry.

simple crystals	rock salt	rutile	zincblende	fluorite	antifluorite
composition	NaCl	TiO_2	ZnS	CaF_2	Mg_2Si
MP entry id	22862	2657	10695	2741	1367
perovskites	cubic	hexagonal	tetragonal	double	Ruddlesden-Popper
composition	$SrTiO_3$	$SrIrO_3$	$CaTiO_3$	$Cs_2AgBiBr_6$	Sr_2TiO_4
MP entry id	5229	17097	3442	1078250	5532

Table A1 Names, compositions and IDs of 5+5 crystals whose invariants are in Fig. 3 and A1.

Fig. A1 The invariants AMD_k and ADA_k from Definition 4 and their weighted versions taking into account atomic masses for five perovskites from MP in Table A1. **Left**: unweighted. **Right**: weighted.

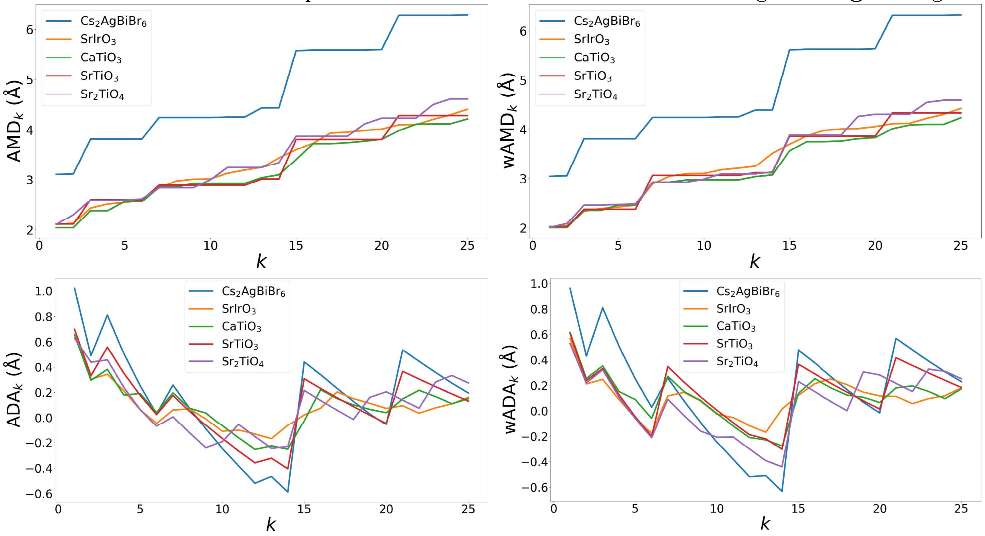

The instructions below will reproduce the distances between the structures in Fig. 5, whose CIFs are publicly available at these URLs:

- icsd_670065.cif: https://www.ccdc.cam.ac.uk/structures/Search?Ccdcid=670065&DatabaseToSearch=Published
- icsd_139006.cif: https://www.ccdc.cam.ac.uk/structures/Search?Ccdcid=139006&DatabaseToSearch=Published
- MnAgO2.cif: Under `Supplementary-Data\Structure_Files` in the supplementary data at https://www.nature.com/articles/s41586-023-06734-w#Sec14

The supplementary materials also include the script `compare_MnAgO2.py` for the distances. With Python 3.9+, install average-minimum-distance (1.5.3) [66] with

```
pip install average-minimum-distance==1.5.3
```

Run the script with

```
python compare_MnAgO2.py
```

The expected output is:

```
>> EMD(PDA(MnAgO2, 100), PDA(icsd_670065, 100)) = 0.0975A
>> EMD(PDA(MnAgO2, 100), PDA(icsd_139006, 100)) = 0.3675A
```

Fig. A2 Distances in Angstroms between 5 simple crystals and 5 perovskites from Table A1. **Upper triangle**: EMDs on weighted wPDA(S; 25). **Lower triangle**: EMDs on unweighted PDA(S; 25).

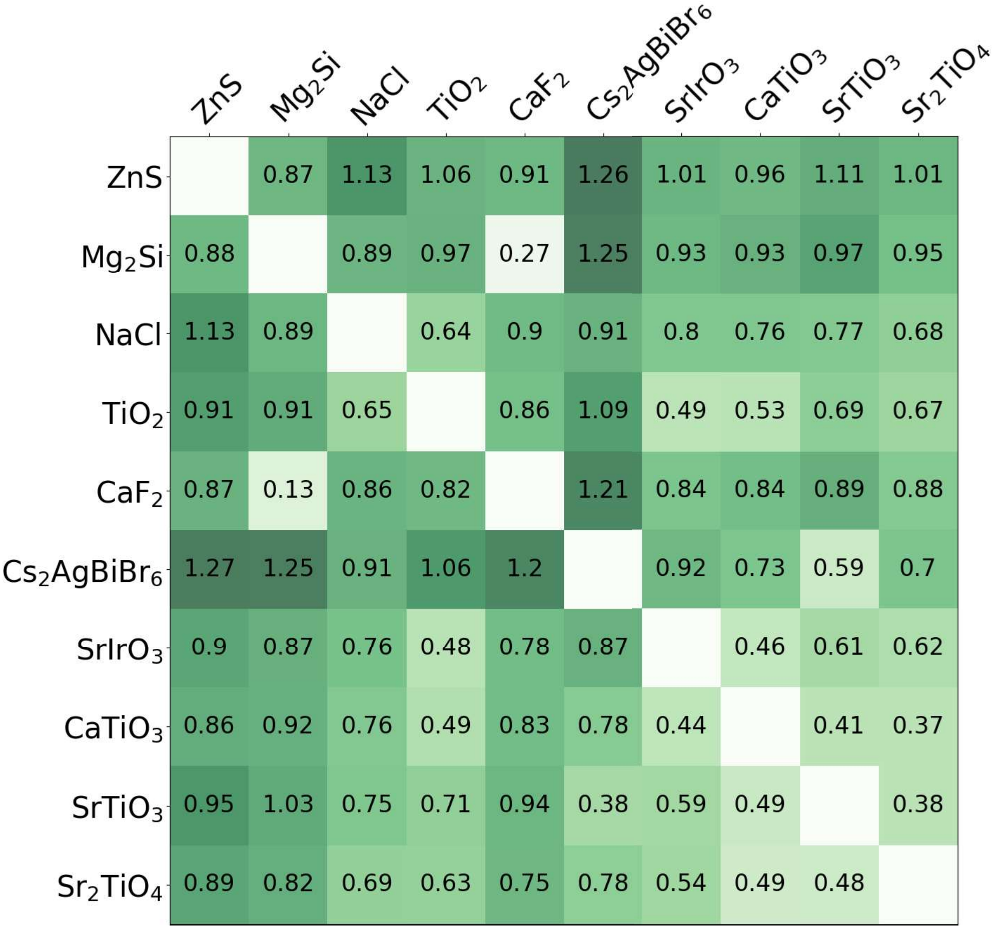

The GNoME paper used the Pymatgen structure matcher [39], which cannot filter out near-duplicate structures according to the quoted steps below.

“1. Given two structures: s1 and s2
2. Optional: Reduce to primitive cells.
3. If the numbers of sites do not match, return False.”

These steps are followed by several heuristic steps which involve finding deviations between atoms in the reduced unit cell. If step 2 above is optionally missed, step 3 can output False (no match) for identical crystals given with different non-primitive cells. If step 2 is enforced, step 3 will output False (no match) for any nearly identical crystals, whose primitive cells can arbitrarily differ due to a tiny atomic displacement as in Fig. 1. For the above reasons, our findings show that this method of comparing structures was insufficient to filter out existing duplicates from materials databases, resulting in the AI silently reproducing near-duplicates from the training set.

Definition 7 can use arbitrary weights of points so that the total weight within a unit cell is 1 after normalization. When all equal rows of PDD(S; k) are collapsed to a single row, the discontinuity in Fig. 1 is properly resolved. The unweighted version of EMD on PDAs below uses zero-sized points at atomic centers with equal weights, which can be multiplied (before normalization) by atomic masses or ionic radii.

Definition 7 (Earth Mover’s Distance EMD [26])**.** Consider any matrix PDA(S; k) as a distribution of rows $R_i(S)$ with weights $w_i(S)$ for $i = 1, \dots, m(S)$ such that $\sum\limits_{i=1}^{m} w_i = 1$. The *Earth Mover’s Distance* $\text{EMD}(\text{PDA}(S;k), \text{PDA}(Q;k)) =$

$\min\limits_{f_{ij}} \sum\limits_{i=1}^{m(S)} \sum\limits_{j=1}^{m(Q)} f_{ij} L_\infty(R_i(S), R_j(Q))$ is minimized for all real $f_{ij} \geq 0$ (called *flows*) subject to the conditions $\sum\limits_{i=1}^{m(S)} f_{ij} \leq w_j(Q)$, $\sum\limits_{j=1}^{m(S)} f_{ij} \leq w_i(S)$, $\sum\limits_{i=1}^{m(S)} \sum\limits_{j=1}^{m(Q)} f_{ij} = 1$.

The first condition $\sum\limits_{j=1}^{m(Q)} f_{ij} \leq w_i(S)$ means that not more than the weight $w_i(S)$ of the component $R_i(S)$ 'flows' into all components $R_j(Q)$ via 'flows' f_{ij} for $j = 1, \dots, m(Q)$. The second condition $\sum\limits_{i=1}^{m(S)} f_{ij} = w_j(Q)$ means that all 'flows' f_{ij} from $R_i(S)$ for $i = 1, \dots, m(S)$ 'flow' into $R_j(Q)$ up to the maximum weight $w_j(Q)$. The last condition $\sum\limits_{i=1}^{m(S)} \sum\limits_{j=1}^{m(Q)} f_{ij} = 1$ forces to 'flow' all rows $R_i(S)$ to all rows $R_j(Q)$.

Proof of Theorem 6. Let S be obtained from a periodic point set $Q \subset \mathbb{R}^n$ by perturbing every point of Q up to Euclidean distance ε, which is smaller than a minimum half-distance between any points of Q. Then S, Q have a common lattice by [67, Lemma 4.1] and hence the same number m of points in a common unit cell, and equal Point Packing Coefficients $\text{PPC}(S) = \text{PPC}(Q)$ from Definition 3.

Since Definition 4 uses the L_∞ metric on rows of PDAs, the Earth Mover's Distance is unaffected by subtracting the same term $\text{PPC}\sqrt[3]{k}$, so $\text{EMD}(\text{PDD}(S;k), \text{PDD}(Q;k)) = \text{EMD}(\text{PDA}(S;k), \text{PDA}(Q;k))$. Then [9, Theorem 4.3] implies that $\text{EMD}(\text{PDA}(S;k), \text{PDA}(Q;k)| \leq 2\varepsilon$. The minimum for all sets Q in a finite dataset D can not be larger, so $\text{LND}(S;D) \leq 2\varepsilon$ by Definition 5.

Conversely, assume that S is obtained from $Q \in D$ by perturbing every atom of Q up to Euclidean distance $\varepsilon < 0.5\text{LND}(S;D) < r(Q)$. The previously proved inequality implies that $\text{LND}(S;D) \leq 2\varepsilon < \text{LND}(S;D)$, which is a contradiction. □

The figures below include high resolution images from Fig. 6.

Fig. A3 A-lab crystals in cyan over the ICSD and MP heatmap in the coordinates (density, ADA_1).

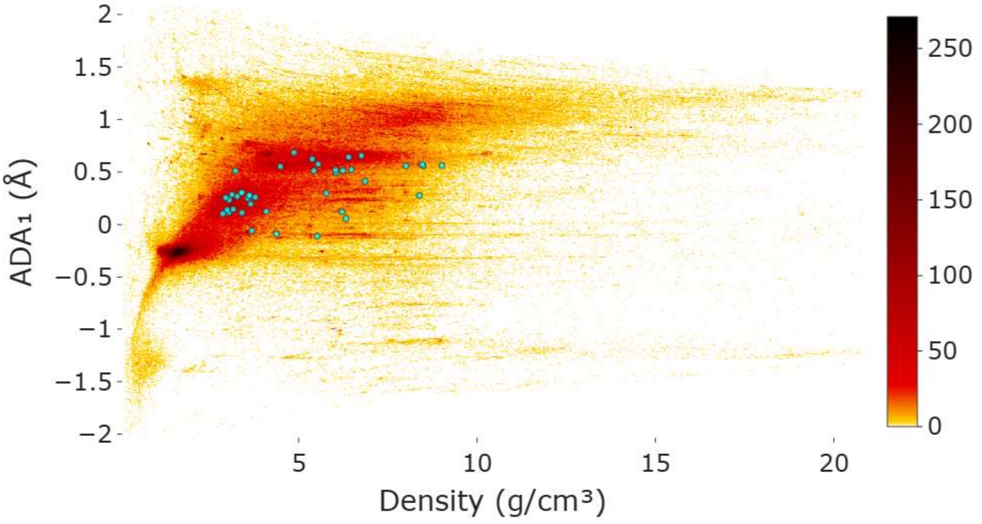

Fig. A4 A-lab crystals in cyan over the ICSD and MP heatmap in the coordinates (ADA_2, ADA_3).

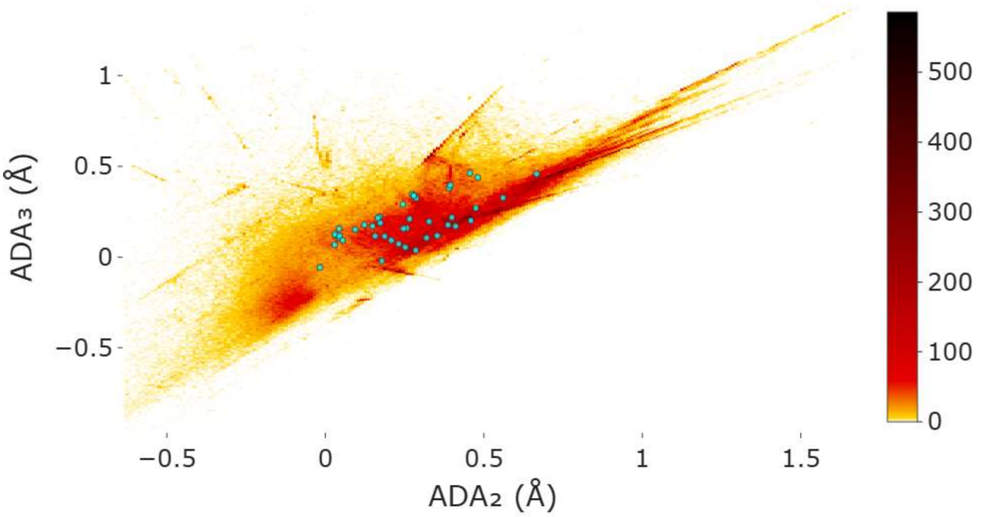

Fig. A5 A-lab crystals in cyan over the ICSD and MP heatmap in the coordinates (ADA_4, ADA_5).

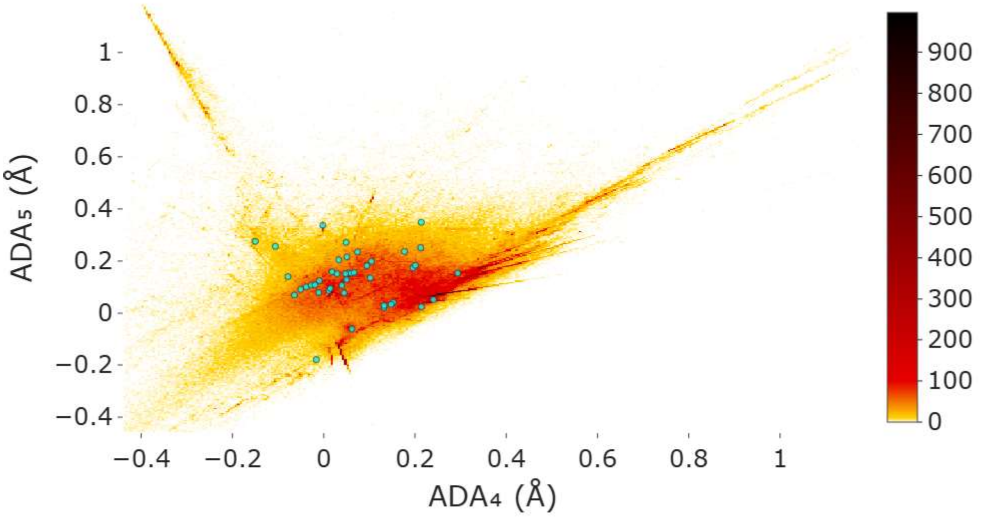

Fig. A6 A-lab crystals in cyan over the ICSD and MP heatmap in the coordinates (ADA_1, ADA_{20}).

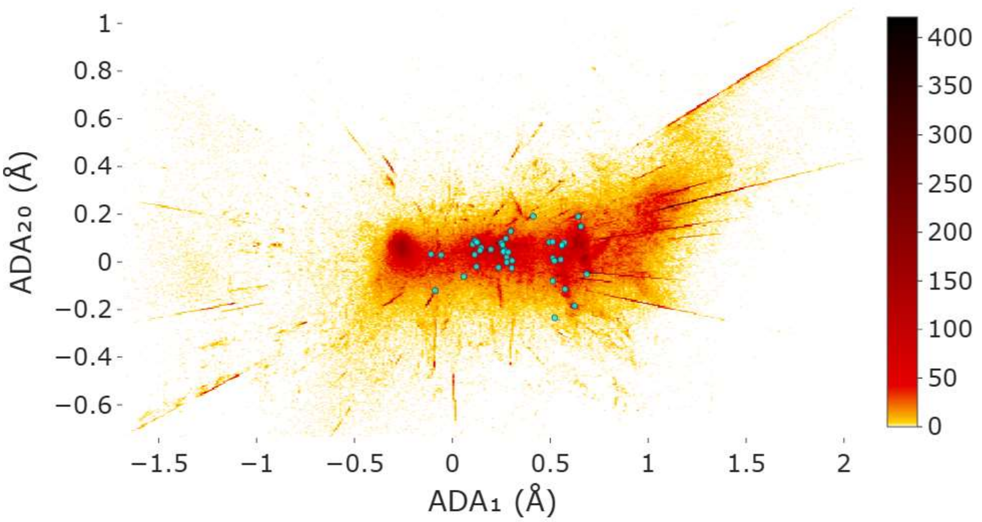

Fig. A7 A-lab crystals over the ICSD and MP heatmap in the coordinates (ADA_{20}, ADA_{100}).

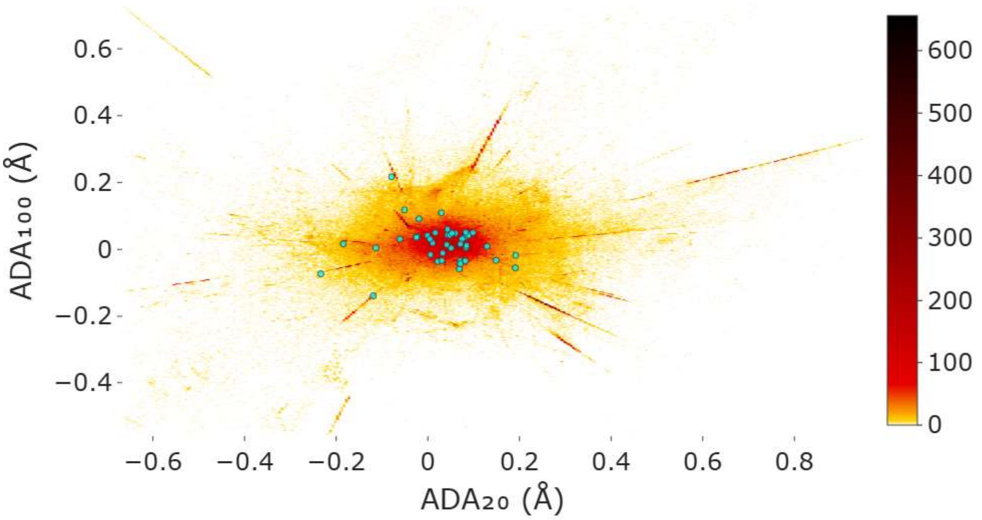

Supplement: Supplementary file 1 — Supplementary Material 1. [file 41598_2025_10672_MOESM1_ESM.pdf]
